# Implementing a federated regional diabetes register in a decentralized health system: implications for healthcare organization and the European Health Data Space

**DOI:** 10.3389/fpubh.2026.1864673

**Published:** 2026-07-16

**Authors:** Fabrizio Carinci, Rossella Messina, Martina Mencarelli, Massimo M. Michelini, Amelia Bici, Arianna Berardo, Alessandra Dei Cas, Elisa Iezzi, Raffaella Aldigeri, Concetta Tania Di Iorio, Stefano Gualdi, Maria Pia Fantini, Riccardo Bonadonna, Nicholas Nicholson, Massimo Massi Benedetti, Paolo Di Bartolo

**Affiliations:** 1 Unicamillus International Medical University, Rome, Italy; 2 AUSL Romagna, Ravenna, Italy; 3 AUSL, Reggio Emilia, Italy; 4 University of Parma, Parma, Italy; 5 University Hospital of Parma, Parma, Italy; 6 Serectrix Srls, Pescara, Italy; 7 Internet Express, Pescara, Italy; 8 University of Bologna, Bologna, Italy; 9 University of Verona, Verona, Italy; 10 AOU, Verona, Italy; 11 European Commission, Joint Research Centre (JRC), Ispra, Italy; 12 Hub for International Health Research, Perugia, Italy

**Keywords:** diabetes, European Health Data Space, federated data systems, health systems, real-world data

## Abstract

**Background:**

Fragmentation of health information systems remains a major barrier to effective health system performance across Europe, particularly in decentralized settings. Federated data infrastructures have been proposed as a scalable solution, but evidence on their implementation at population level remains limited.

**Objectives:**

We aimed to describe the implementation of a federated information infrastructure at regional level and assess the completeness and variability of linked data across participating centers, discussing implications of scaling up the approach within the European Health Data Space.

**Materials and methods:**

We carried out a population-based cohort study linking administrative, clinical, and patient-reported data across three healthcare organizations in Emilia-Romagna, Italy, covering over 2.1 million residents. Data were analyzed using a federated architecture without sharing individual-level information. Baseline characteristics were assessed at 1 January 2019, with longitudinal follow-up over 6 years.

**Results:**

We identified 116,552 individuals with diabetes (prevalence 5.6%). Among individuals with available classification, 90.8% had type 2 diabetes. Clinical data were available for 43.5% of patients in charge of diabetes clinics, with substantial heterogeneity across centers (12.8–80.6%). Among those with clinical data, 27.5% had baseline HbA1c levels below 48 mmol/mol, while 69% had elevated systolic pressure (≥130 mmHg) and 56.6% had high diastolic pressure (≥80 mmHg). Sociodemographic variables were largely missing. Patient-reported outcomes were collected in 521 individuals, demonstrating feasibility but limited scalability.

**Discussion:**

Federated linkage of administrative, clinical, and patient-reported data is feasible at regional scale and enables population-level monitoring of diabetes care. However, variability in data completeness was primarily driven by organizational and governance factors rather than technical capacity. These findings provide empirical evidence that strengthening health data systems requires alignment of healthcare organization and service delivery models, beyond technical solutions alone.

**Conclusion:**

The REWINDER project built a collaborative information infrastructure using federated linkage of different data sources and person-reported outcomes, independently managed by local healthcare organizations. The project has made available a large database to inform policy and planning of diabetes care across the region. The system may be used as a model that can be conveniently scaled up to other geographical areas and chronic diseases.

## 1 Introduction

Unified health information systems are essential to support clinical decision-making, health system governance, and population-level planning, particularly in the context of chronic diseases. However, across Europe, the effective use of real-world data remains constrained by fragmentation of data sources, limited interoperability, and heterogeneous governance structures (1).

Modern technological solutions can unify existing systems and information infrastructures to achieve connectivity and interoperability to deliver an increasing range of services, using different hardware devices and overcoming many of the existing technological challenges. Unified systems exploit data interoperability by using data linkage across multiple sources, systematically and consistently over time (2).

Challenges are particularly evident in decentralized healthcare systems, where multiple administrative and clinical entities operate with varying degrees of autonomy.

Italy represents a paradigmatic example of such complexity. While the National Health Service (Servizio Sanitario Nazionale, SSN) guarantees universal coverage, the organization of care across regions and local healthcare authorities has led to substantial variability in data availability, accessibility, and integration (3, 4). In response to these issues, new e-health initiatives, e.g., the Italian Electronic Health Record (Fascicolo Sanitario Elettronico—FSE) have been promoted by the Ministry of Health (5). However, their implementation is not widespread (6).

Administrative data in Italy are relatively standardized and have been widely used for epidemiological and health services research (7). However, their clinical depth is limited, and linkage with richer clinical datasets remains inconsistent. At the same time, clinical data collected within specialist services are often not systematically integrated into broader population-based systems. Patient-reported outcome measures (PROMs), increasingly recognized as a key component of person-centered care, are not captured in routine practice (8, 9).

At European level, recent initiatives such as the European Health Data Space (EHDS) and the Joint Action on Cardiovascular Diseases and Diabetes (JACARDI) have explicitly highlighted the need for integrated, interoperable, and scalable health data infrastructures to support policy-making and improve outcomes in non-communicable diseases (NCDs) (10). However, while these initiatives provide strategic frameworks and recommendations, evidence on their practical implementation at population level remains limited. These initiatives require not only technical interoperability, but also alignment of organizational and governance structures to ensure meaningful use of health data.

Federated data infrastructures have emerged as a promising approach to address these challenges. In federated systems, data remain under local control but are analyzed through shared standards and common methodologies, allowing comparability while preserving data privacy (11, 12). This model is particularly well suited to decentralized systems, as it leverages existing organizational structures rather than attempting full centralization.

Diabetes represents an ideal use case for testing such infrastructures. It is a highly prevalent chronic condition requiring long-term, coordinated care across multiple providers, and its management relies on the integration of clinical indicators, service utilization data, and PROMs. International initiatives, including the International Consortium for Health Outcomes Measurement (ICHOM) standard set, have defined relevant outcome measures, but their implementation in routine practice remains limited (13, 14).

Within this context, the REWINDER project (“Real-World data and patient-reported outcomes IN Diabetes”) was designed to develop and operationalize a federated regional infrastructure linking administrative, clinical, and patient-reported data across multiple healthcare organizations in the Emilia-Romagna region (15).

In this paper, we will present the information infrastructure of the project, with the following aims:

- to describe in detail the approach adopted in REWINDER to collect, link and analyze federated databases
- to report the general characteristics of the study population and the variability of practices across participating units and territories.

The presentation of the results will allow drawing reflections regarding the potential implications of using the proposed approach for other applications at national and international level, within the federated ecosystem of the European Health Data Space.

## 2 Materials and methods

### 2.1 Study design and setting

We conducted a population-based cohort study within REWINDER, including residents of three large healthcare organizations in the Emilia-Romagna region (AUSL Romagna, AUSL Reggio Emilia, and AOU Parma).

The study protocol of REWINDER has been already described in detail elsewhere (15). The study combined retrospective longitudinal analysis of routinely collected data with prospective collection of PROMs. The reference date (time zero, denoted as t_o_) was set at 1 January 2019, with longitudinal follow-up extending up to 6 years. The study was authorized by all relevant ethical committees (15). The research project started on 27 April 2022 and ended after 36 months.

In this report, diabetes was used as a tracer condition to test system-level data integration across multiple sources.

### 2.2 Study population

At baseline, the total number of people with diabetes (PWD) in Emilia-Romagna identified by the regional government was equal to 273,968 over a total population of 4,471,485, corresponding to a prevalence of 6.1% (16).

The three areas participating in REWINDER accounted for almost half of the population in the region, adding up to 2,104,342 individuals. The study population included all PWD alive and resident in the study areas at baseline, identified using a validated regional algorithm based on administrative data sources, including hospital discharge records, drug prescriptions, and disease-specific exemptions from co-payment. This approach allowed consistent identification of cases across participating centers and ensured population-level coverage.

### 2.3 Definition of the federated data infrastructure

The federated model was designed to enable data linkage and analysis while preserving local data governance and complying with data protection regulations.

The fundamental criteria of “privacy-by-design” and “federated architecture” were used to carry out a series of collaborative steps, including: definition of the clinical problem, selection of data sources, reporting templates, privacy impact assessment, system architecture, data dictionary, software development, federated analysis, technology transfer and evaluation.

More details about the implementation model are included in Table 1.

**Table 1:** Federated model of the REWINDER study.

Area	Description
Overarching principles	
Privacy-by-design	The project adopted the criteria of the “BIRO approach” adopted by the federated EUBIROD network (11, 42, 43) in full compliance with the General Data Protection Regulation (GDPR) (33, 34).
Federated architecture	The project implemented a federated architecture for the secure analysis of the study cohort, without exchanging individual data across sites.
Steps used for implementation*	
Definition of the clinical problem	The clinical problem was discussed in a series of calls and two in-person meetings held at the coordinating center in Ravenna, including representatives of all participating units.
Assessment of data sources	The project team agreed CDEs of the ICHOM standard set to be extracted from local administrative datasets, clinical datasets and the data collection of PROMs.
Agreement of a common reporting template	A common reporting template was identified by the project team to deliver a simple dashboard available for all centers
Privacy impact assessment	A privacy impact assessment was carried out independently by each local healthcare organization, involving Data Protection Officers (DPOs) and Data Governance Administrators (DGAs), to agree on the specific information infrastructure adopted in each case (see Supplementary Figure S2)
Selection of the best information architecture	The information infrastructure of REWINDER was defined in a number of meetings. DPOs and DGAs were involved in the selection of the encryption method (SHA256) used to generate a pseudonym (CFSHA256) from the tax file number (“Codice Fiscale”) used by the SSN as the official unique personal identifier (UPI). Participating centers could process own data, using assigned credentials to log in a centralized server using secure Remote Desktop Protocol (RDP) via VPN. The server, maintained by the ICT systems of AUSL Romagna, included all REWINDER software properly installed for independent use.
Metadata of common data elements and standardized indicators	Metadata of CDEs were defined and all technical specifications for tables included in REWINDER were shared to contributing data holders for the extraction of all tables (see Supplementary data for all details).
Development of software for database integration and federated analytics	Software for database integration and federated analytics was progressively developed at local and central level. Any discrepancies between local tables and standard items were mapped against common specifications in a data translation routine included in NeuBIRO (see below).
Data collection and analysis	Data collection and analysis was collaboratively performed. All administrative tables were included in the database “Adm-DB.” Clinical information was included in the database “Clin-DB” formed by all diabetes clinics within each organization. The PROMS table included questionnaires collected on paper by doctors and nurses involved in the study, digitized using different means (see below).
Technology transfer	The experience of each local healthcare organization was shared among members of the network. The common know-how may now be transferred to other parts of the region.
Evaluation	The regional government received reports and evaluated the project

*Adopted from the BIRO approach (43).

### 2.4 Common data model

Three main types of data sources were included in a common data model to capture complementary dimensions of diabetes care, including service utilization, clinical status, and patient experience.

The characteristics of clinical outcomes, risk factors, additional case-mix variables and PROMs included in REWINDER have been presented in detail in the study protocol (15).

Briefly, the common data model included the following data sources:

- Administrative data, including demographic information (Supplementary Table S-L1), hospitalizations (Supplementary Tables S-L2–S-L4), admissions to the emergency departments (Supplementary Table S-L5) and pharmaceutical prescriptions (Supplementary Table S-L6), information on COVID-19 (Supplementary Table S-L7).
- Clinical data, extracted from electronic records of diabetes clinics, including laboratory measurements and clinical parameters (Supplementary Tables S-L8A/B).
- PROMs, collected prospectively through three standard questionnaires (15) administered during clinical visits

The model also included a subset of derived variables for which repeated measurements were collapsed into annual summaries, to facilitate statistical analysis (Supplementary Tables S-R1–S-R6).

The structure of the common data model is presented in Supplementary Figure S1 of supplementary data. The supplementary materials also include the complete data dictionary for all common data elements (CDEs). Coding of CDEs has been based on the global ICHOM standard set for diabetes (8).

### 2.5 Construction of the longitudinal cohort

**Figure 1:**
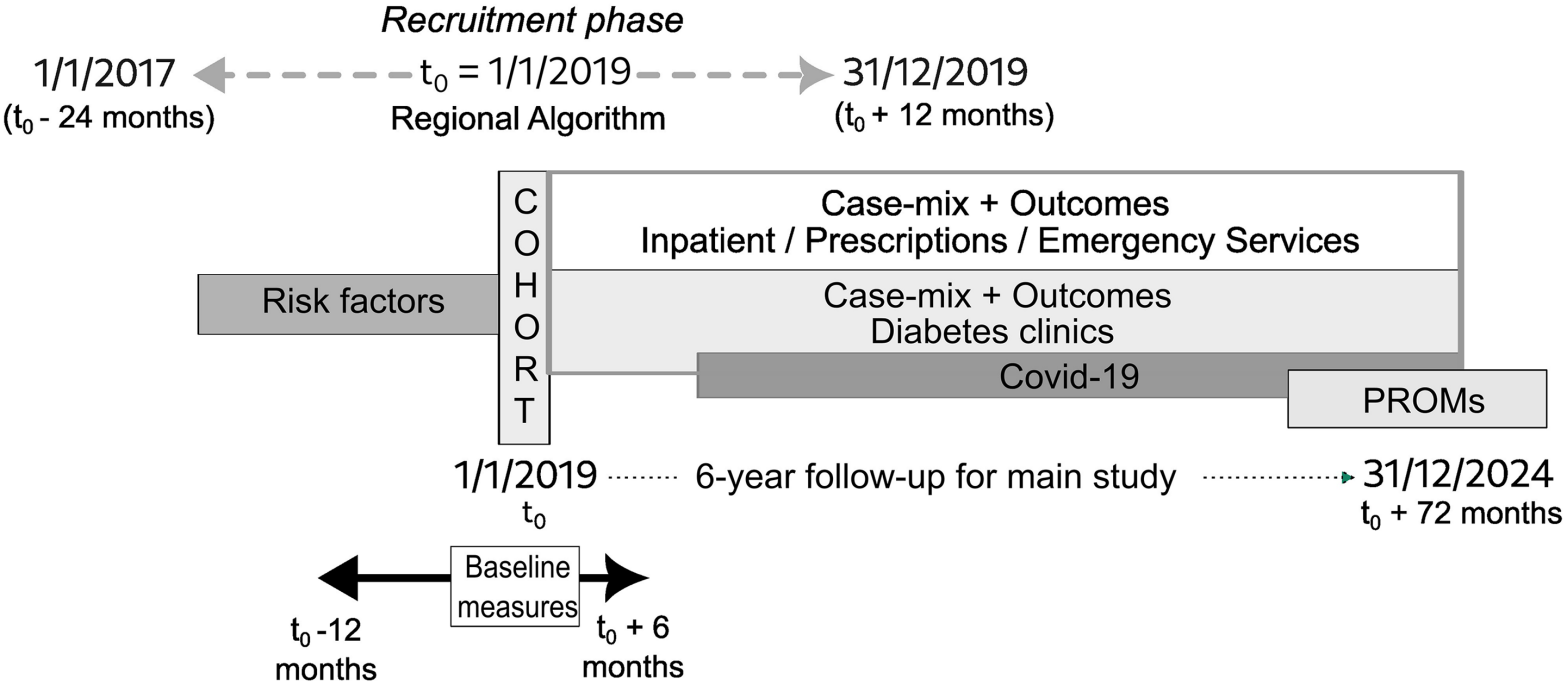
Longitudinal design of the REWINDER study.

The procedure applied for the construction of the REWINDER longitudinal cohort is described in detail in Figure 1.

A standardized algorithm was applied for case-identification, using linked administrative data for the years 2017–2019. The timeframe was selected as the “recruitment phase” used by the algorithm for the interval: t0–24 months, t0 + 12 months (see dashed double-sided arrow in the upper part of Figure 1). The case-identification algorithm has been described in detail in the study protocol (15).

The algorithm successfully identified the reference cohort of REWINDER, including people with diabetes alive and resident in any of the target areas on 1st January 2019.

Subsequently, all valid administrative and clinical data recorded between 2017 and 2019 were extracted retrospectively to collect risk factors and comorbidities. The database was completed by including all records available from administrative and clinical data for the reference cohort for the ensuing 6 years (see dotted arrow at the bottom of Figure 1).

Baseline measures for clinical variables were taken as the closest valid measurement available between t_0_–12 months and t_0_ + 6 months (see left and right solid arrows at the bottom of the cohort in Figure 1).

Clinical complications during follow-up were considered as primary outcomes of the study among subjects enrolled in the cohort.

The sample of individuals with PROMs measurements was not aligned with the main follow-up, as the data were prospectively collected by participating units at different times across 2024–2025.

For this sample, we considered the correlation between changes in glycated hemoglobin (HbA1c) and PROMs levels, measured at 0–6 months, as a secondary outcome.

### 2.6 Data standardization and processing

The steps adopted for data standardization and statistical analysis are shown in Supplementary Figure S2.

Different roles were involved in carrying out these procedures, including data protection officers (DPOs), data governance administrators (DGAs), IT Providers and consultants, in addition to members of the project team.

The processing of administrative data was harmonized across centers through the national definitions. The technical specifications were translated into SQL queries by local data managers.

Clinical data were extracted from the databases maintained in diabetes clinics of participating centers, all sharing the same software adopted by the National Association of Medical Diabetologists (AMD) (17).

All records available before and after the recruitment period for “active patients” were included in the clinical tables. The operational definition of “active patients” referred to people with a recorded diagnosis of diabetes (type 1, type 2 or other) and at least one recorded clinical assessment (measurement of weight, systolic or diastolic blood pressure) during the observation period 2017–2019 (18).

Data were pseudonymized locally using a common encryption procedure (SHA256) applied to the unique personal identifier (UPI). The procedure generated a CDE called CFSHA256. Each participating center retained control over own data, by processing records locally using shared definitions and standardized analytical procedures.

However, the methods used by centers to extract clinical tables were different.

The units of Romagna and Parma developed an *ad hoc* tool to translate the AMD export XML dataset into the specified REWINDER format. However, the personal ID adopted for clinical data was internally coded. To generate the common pseudonym, the developers of Metaclinic produced a hash table matching internal IDs with the UPI, which was later encrypted into the CFSHA256 by staff of the center, adding it to the tables exported by the REWINDER tool.

The entire process was skipped by Reggio Emilia, which directly mapped the Metaclinic database to the requested format of clinical data. The method required direct processing of the proprietary database, which was beyond the capacity of other centers.

PROMs were collected from eligible individuals signing an informed consent to participate in the study. PWD filled three questionnaires on paper format, with the support of nurses and members of the project team, while attending a visit at a diabetes clinic. The original UPI was assigned to each respondent both at baseline and follow-up. Interviews at follow-up were carried out by Romagna mostly by telephone.

The digitization of the questionnaires was carried out by participating centers using different methods. Romagna and Reggio-Emilia used their applications of choice (“Health Meeting” and “Smarty,” respectively), while Parma preferred data entry in an Excel sheet. In all cases, data were exported into csv files, after replacing the UPI with the pseudonym. These operations were performed by members of the project team.

Following digitization, data quality checks were performed to evaluate the consistency of PROMs with the protocol specification. The overlap between the three main data sources used in REWINDER highlighted cases of misalignment: (a) a patient could be diagnosed in the time lag between the recruitment phase and the administration of the questionnaires; (b) patients in the PROMs cohort may have not been classified as “active patients,” due to the list not being available at the start of interviews; (c) follow-up interviews carried out by phone may have not been associated to any clinical data.

In addition, follow-up measures were rarely taken at precise time intervals, with recordings spanning weeks or even months around the expected timeframe of 6 months. This situation caused incomplete overlap of administrative, clinical and PROMs data at both baseline and follow-up.

In the end, only PROMs referred to subjects compliant with the protocol, linked with clinical data, and with both valid baseline and follow-up measurements, were considered eligible for the main analysis of the study.

**Figure 2:**
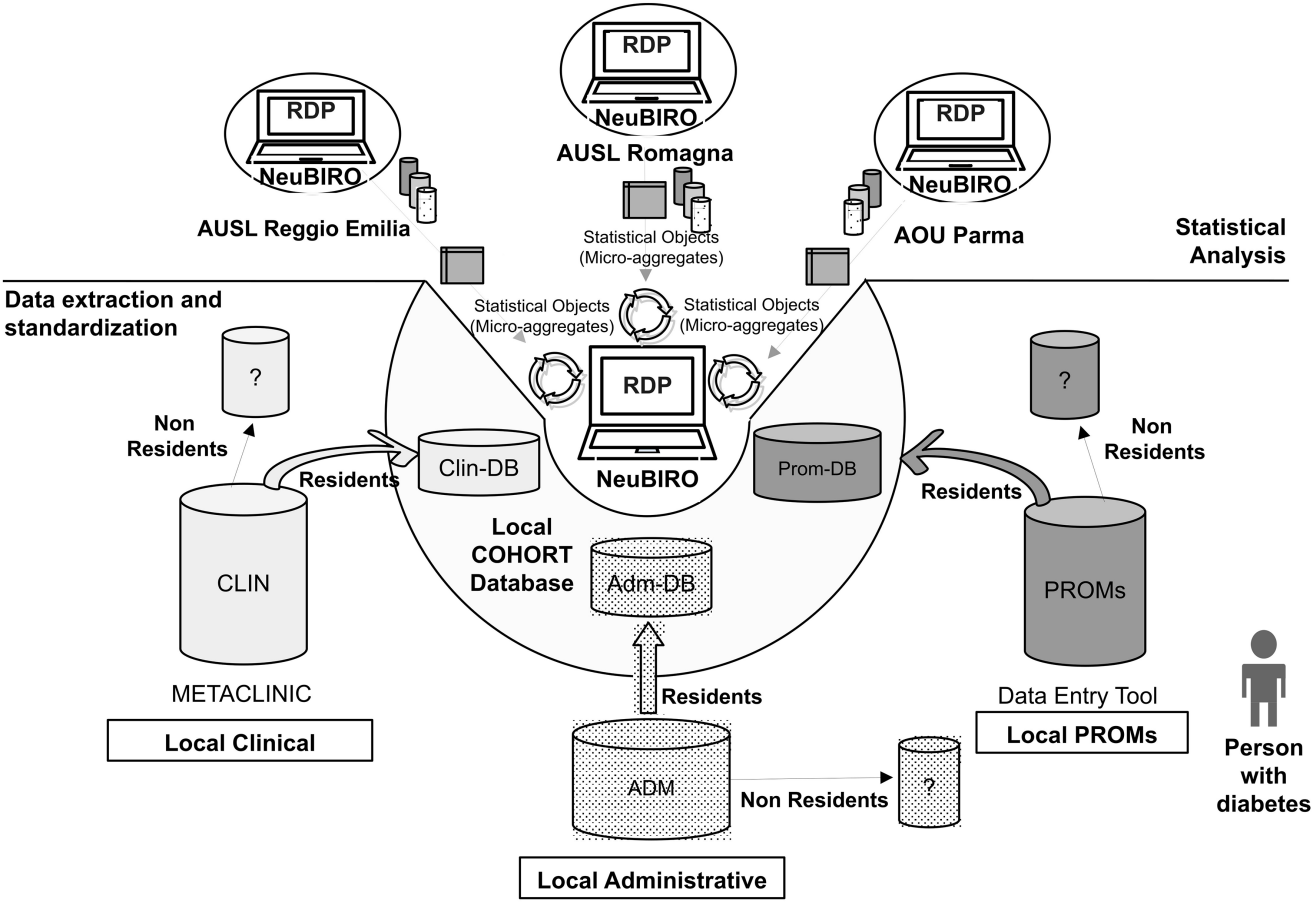
Diagram of the REWINDER information infrastructure. Database integration. Data sources involved in data collection: CLIN, Clinical data from MetaClinic system used in diabetes clinics; Clin-DB, Clinical database containing patient data from individual ICT centers; Adm-DB, Administrative database with patient demographics and healthcare records; PROMs databases, Patient-Reported Outcome Measures. Data tools, Light Gray Arrow, Data flows related to clinical data management, using specific tools; Dark Gray Arrow, Data flows related to PROMs recruitment and integration; Links clinical records and PROMs data entered through specific tools. NEUBIRO STATISTICAL SYSTEM. RDP, Secure Remote Desktop Protocol to access NeuBIRO from ICT systems located at AUSL Romagna, AUSL Reggio Emilia and AO Parma; NeuBIRO Central Server, Centralized data repository for the Rewinder Cohort Database, integrating data from clinical and administrative sources; Statistical Objects (Micro-aggregates), Processed or anonymized statistical summaries used for data aggregation across regions. ICONS AND SYMBOLS. Database Cylinders, Represent data storage systems (e.g., clinical, administrative, PROMs); Person icon, Represents patients participating in PROMs; Non-Residents, Indicates external or unknown data sources outside the local health authority system.

The final federated information infrastructure is presented in Figure 2.

All administrative, clinical data and PROMs were stored in a reserved location of the REWINDER server, available for use of NeuBIRO by each collaborating center, to carry out the statistical analysis.

### 2.7 Federated statistical analysis

The present report is limited to the description of baseline characteristics of the study cohort, assessed at 1st January 2019.

Federated analysis was conducted through a distributed approach implemented in the NeuBIRO software, whereby aggregated results (rather than individual-level data) were shared and combined centrally. This approach was based on the EUBIROD framework and aligned with privacy-by-design principles.

The general features of NeuBIRO have been described in detail in the study protocol (15). The functions specifically implemented in REWINDER are presented in Table 2. The NeuBIRO software is freely available as open source (19).

**Table 2:** Functions of the NeuBIRO software(19).

Function	Description
Configuration	The descriptors of items in each data source are customized to import columns from local formats to the CDEs
Data import and quality check	Using the configuration files, local data are imported into a standardized database
Local statistical processing	SQL queries are used to extract and aggregate selected records from the internal database as input for local analysis. Outputs were aggregated (“statistical objects”) to produce local reports
Transfer to the coordinator folder	Aggregate data are moved to a shared server location with a log of the original processing
Central statistical processing	Statistical objects are used by R routines to process centers’ data and deliver global results

Descriptive analyses were used to characterize the study population and assess variability across centers. Categories chosen for the variables and thresholds for categorization were consistent with ICHOM definitions (8). Results were presented as absolute and relative frequencies, stratified by participating center to explore differences in data availability and clinical characteristics.

Data completeness was evaluated for each variable and data source. Missing values were reported as “population-based” as they were referred to the overall cohort. The percentage of missing values attributable to a specific data source can be calculated as the difference between the percentage of missing “population-based” and the percentage of records extracted from administrative data.

Demographic and socio-demographic characteristics included:

- Sex (Male, Female);
- Age classes in years (0–15, 16–44, 45–64, 65–84, ≥ 85);
- Education level (classified as None, Primary, Secondary or Tertiary);
- Smoking status (No, Yes, Ex-smoker);
- Physical activity (No, Yes)

Clinical conditions included:

- Years since diabetes diagnosis (categorized as <1, 1–5, 6–15, >15);
- Type of diabetes (Type 1, Type 2, Other Types)

Clinical measurements included:

- HbA1c, categorized using mmol/mol (low: < 48, mid: ≥ 48 to < 58, vs. high: ≥ 58) or % (low: < 6.5, mid: ≥ 6.5 to <7.5, vs. high: ≥ 7.5). Due to recordings made with either one or both units, valid values were translated as needed, into both mmol/mol and %, using the available DCCT/IFCC–NGSP master equations.^1^
- Body Mass Index (BMI, kg/m^2^), categorized as underweight (<18.5), normal (≥ 18.5,<25), overweight (≥ 25,<30), or obese (≥ 30);
- Systolic Blood Pressure (classified as Low-mid: <130, vs. High: ≥130 mmHg);
- Diastolic blood pressure (classified as Low-mid: < 80, vs. High: ≥ 80 mmHg).

PROMs were reported as the total number of people with diabetes who filled questionnaires at baseline. In addition, we reported the absolute and relative frequencies of those who were lost to follow-up vs. those with a valid follow-up between 4 and 10 months. These figures were reported to outline the feasibility of data collection of PROMs. The psychometric characteristics and variability of scores will be presented in a dedicated paper.

Given the heterogeneity in data sources and governance structures, analyses were designed to provide system-level insights rather than causal inference. All analyses were conducted using standardized procedures within the federated infrastructure, ensuring comparability across centers, using NeuBIRO and the R language (20).

## 3 Results

### 3.1 Cohort composition

A total of 116,552 persons with diabetes were included in the study cohort, corresponding to a prevalence of 5.6% for the three geographical areas of interest.

**Figure 3:**
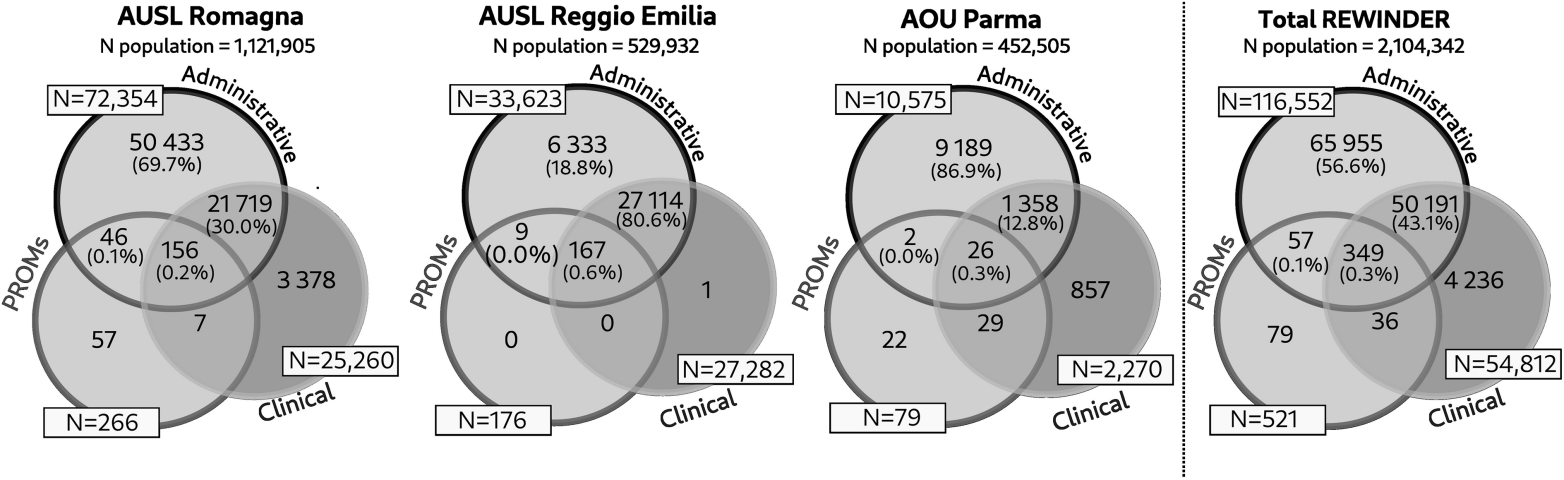
People in diabetes in REWINDER databases by center and source. *N for each center are referred to the total number of subjects by source. Numbers in circles are N subjects for each subset (% shown only for 2019 cohort). Clinical data are referred to the value closest to 1st Jan 2019 between (1st Jan 2018 to 30th Jun 2019), for patients active between 2017 and 2019 any diabetes diagnosis with at least one recorded value of weight, systolic/diastolic blood pressure.

The composition of the overall database of the project, presenting the overlap between different data sources, is presented in Figure 3.

The majority of cases identified were resident in Romagna (*N* = 72,354, corresponding to 62% of the study cohort and 6.4% prevalence in the area), while nearly one third in the province of Reggio-Emilia (*N* = 33,623, 29% of the cohort, 6.3% prevalence), and one fifth in the area of Parma (*N* = 10,575, 19% of the cohort, 2.3% prevalence).

### 3.2 Data availability and heterogeneity

Clinical data were available for 43.5% in charge of diabetes clinics as “active patients” in the reference period. There was a substantial variability of this coverage across centers, ranging from 12.8% in Parma to 80.6% in Reggio Emilia, with an intermediate coverage of 30.0% for Romagna. Almost complete baseline profiles were available for parameters of direct interest for care provision, e.g., HbA1c, BMI and blood pressure. The distribution of these characteristics could be observed across participating units.

Sociodemographic and behavioral variables were incompletely captured. Missing data at population level reached 95.8% for educational level, 81.0% for smoking status, and 84.6% for physical activity. However, some variability was observed across centers, with Reggio Emilia showing higher completeness for lifestyle variables compared to other sites (smoking status and physical activity missing in 68.3 and 72.4% respectively).

### 3.3 General characteristics

The descriptive results of the study cohort measured at baseline are shown in Table 3.

**Table 3:** Baseline characteristics of the REWINDER cohort as of January 1st, 2019*.

Variable	AUSL Romagna		AUSL Reggio-Emilia		AOU Parma		Total	
	N	%	N	%	N	%	N	%
N (population-based)	72,354	100.0	33,623	100.0	10,575	100.0	116,552	100.0
Clinical records	21,875	30.2	27,281	81.1	1,384	13.1	50,540	43.4
Sex								
Male	38,428	53.1	18,498	55.0	6,193	58.6	63,119	54.2
Female	33,926	46.9	15,125	45.0	4,382	41.4	53,433	45.8
Age group (years)								
0–15	258	0.4	133	0.4	64	0.6	455	0.4
16–44	3,622	5.0	2,181	6.5	557	5.3	6,360	5.5
45–64	18,941	26.2	9,716	28.9	2,637	24.9	31,294	26.8
65–84	39,790	55.0	17,558	52.2	5,928	56.1	63,276	54.3
≥ 85	9,743	13.5	4,035	12.0	1,389	13.1	15,167	13.0
Years since diabetes diagnosis								
<1	6,100	8.4	2,237	7.1	2,319	21.9	10,656	9.3
1–5	31,458	43.5	7,804	24.9	7,311	69.1	46,573	40.8
6–15	25,360	35.1	14,368	45.9	527	5.0	40,255	35.2
>15	9,436	13.0	6,883	22.0	418	4.0	16,737	14.7
*Missing*	*-*	*-*	*2,331*	*6.9*	*-*	*-*	*2,331*	*2.0*
Type of diabetes								
Type 1	1,338	6.1	1,322	4.8	80	5.8	2,740	5.4
Type 2	19,561	89.6	25,259	92.6	1,059	76.5	45,879	90.8
Other types	976	4.5	700	2.6	245	17.7	1921	3.8
*Missing (population-based)*	*50,479*	*69.8*	*6,342*	*18.9*	*9,191*	*86.9*	*66,012*	*56.6*
Educational level**								
None	25	1.0	15	1.1	15	1.4	55	1.1
Primary	1,656	68.4	962	70.5	637	60.1	3,255	67.1
Secondary	634	26.1	331	24.2	321	30.3	1,286	26.5
Tertiary	110	4.5	57	4.2	87	8.2	254	5.3
*Missing (population-based)*	*69,929*	*96.6*	*32,258*	*95.9*	*9,515*	*90.0*	*111,702*	*95.8*
Smoking status**								
No	5,372	50.2	5,811	54.5	413	50.1	11,596	52.3
Yes	2,314	21.6	1950	18.3	187	22.7	4,451	20.1
Ex smoker	3,020	28.2	2,892	27.2	224	27.2	6,136	27.6
*Missing (population-based)*	*61,648*	*85.2*	*22,970*	*68.3*	*9,751*	*92.2*	*94,369*	*81.0*
Physical activity**								
No	2,244	28.2	3,834	41.3	287	38.8	6,365	35.4
Yes	5,723	71.8	5,453	58.7	453	61.2	11,629	64.6
*Missing (population-based)*	*64,387*	*89.0*	*24,336*	*72.4*	*9,835*	*93.0*	*98,558*	*84.6*
HbA1c mmol/mol (%)***								
< 48 (< 6.5)	3,788 (2784)	22.0 (16.2)	7,907 (6402)	31.1 (25.2)	381 (328)	31.1 (26.8)	12,076 (9514)	27.5 (21.7)
≥ 48, < 58 (≥ 6.5, <7.5)	5,275 (6279)	30.6 (36.4)	7,988 (9493)	31.5 (37.4)	361 (414)	29.5 (33.8)	13,624 (16186)	31.1 (36.9)
≥ 58 (≥ 7.5)	8,170 (8170)	47.4 (47.4)	9,492 (9492)	37.4 (37.4)	482 (482)	39.4 (39.4)	18,144 (18144)	41.4 (41.4)
*Missing (population-based)*	*55,121*	*72.3*	*8,236*	*24.5*	*9,351*	*88.4*	*72,708*	*62.4*
Body mass index (kg/m^2^)***								
Underweight (<18.5)	129	0.8	150	0.8	11	1.0	290	0.8
Normal (≥ 18.5, <25)	3,398	21.0	3,964	19.9	206	18.2	7,568	20.3
Overweight (≥ 25, <30)	5,929	36.6	7,472	37.6	443	39.1	13,844	37.2
Obesity (≥ 30)	6,742	41.6	8,296	41.7	474	41.7	15,512	41.7
*Missing (population-based)*	*56,156*	*77.6*	*13,741*	*40.9*	*9,441*	*89.3*	*79,338*	*68.1*
Systolic blood pressure (mmHg)***								
< 130	4,335	32.3	6,032	30.3	328	28.4	10,695	31.0
≥ 130	9,093	67.7	13,893	69.7	827	71.6	23,813	69.0
*Missing (population-based)*	*58,926*	*81.4*	*13,698*	*40.7*	*9,420*	*89.1*	*82,044*	*70.4*
Diastolic blood pressure (mmHg)***								
<80	6,298	46.9	8,304	41.7	382	33.1	14,984	43.4
≥80	7,132	53.1	11,618	58.3	772	66.9	19,522	56.6
*Missing (population-based)*	*58,924*	*81.4*	*13,701*	*40.7*	*9,421*	*89.1*	*82,046*	*70.4*

*Categories presented in italic refer to missing data (see section 2.7 for related calculations). % calculated on the total of non-missing data; categories presented in italic refer to missing data (see section 2.7 for related calculations); **Most recent non missing value available; ***Clinical data referred to the value closest to 1st Jan 2019 between (1st Jan 2018 to 30th Jun 2019), for patients active between 2017 and 2019 any diabetes diagnosis with at least one recorded value of weight, systolic/diastolic blood pressure.

The distribution of the population by age appeared to be balanced across geographical areas. Overall, over 50% of the population was aged between 65 and 84 years (54.3%) and most cases were males (54.2%).

The duration of diabetes was almost completely recorded as the oldest date available from linked administrative and clinical data, being <1 year in 9.3% cases, 1–5 years in 40.8%, 6–15 years in 35.2% and >15 years in 14.7%, respectively.

The most frequent type of diabetes available from clinical records was type 2 (90.8%), followed by type 1 (5.4%) and other types (3.8%).

Relevant differences were noted between centers, which may reflect heterogeneous patient case mix (e.g., age, disease duration and treatment complexity), timeliness of diabetes diagnosis at origin and referral patterns across centers.

### 3.4 Socio-demographic characteristics

These features were derived entirely from clinical data.

The education level of the cohort was mainly primary (67.1%), followed by secondary (26.5%). Parma had double the number of PWD with tertiary education (8.2%), compared to Romagna (4.5%) and Reggio Emilia (4.2%).

Smoking status was generally balanced, with half of the persons who never smoked (52.3%), as opposed to 20.1% still smoking and 27.6% ex-smokers.

The percentage of people doing physical activity was almost two thirds (64.6%), a percentage that was slightly higher in Ravenna (71.8%), compared to Parma (61.2%) and Reggio Emilia (58.7%).

### 3.5 Clinical characteristics

Among patients with available clinical data, key indicators of disease control showed varied patterns.

HbA1c values were distributed as follows: 27.5% below 48 mmol/mol, 31.1% between 48 and 58 mmol/mol, and 41.4% ≥ 58 mmol/mol.

Differences across centers were observed, with Romagna showing a higher proportion of poorly controlled patients, and Reggio Emilia a comparatively lower proportion.

BMI indicated that most patients were overweight or obese (78.9%). Elevated systolic blood pressure was present in 69.0% of patients, while 56.6% had diastolic blood pressure ≥80 mmHg, a percentage that was slightly higher in Parma (66.9%).

Overall, these clinical indicators were relatively consistent across centers, despite differences in data availability.

### 3.6 PROMs feasibility and follow-up

The main features of PROMs data collection are presented in Table 4.

**Table 4:** Summary of PROMs data collection.

Variable	AUSL Romagna		AUSL Reggio-Emilia		AOU Parma		Total	
	N	%	N	%	N	%		
Baseline (N)	266	51.0	176	33.8	79	15.2	521	100.0
Lost to follow-up	21	7.9	62	35.2	6	7.6	89	17.1
Patients with a valid follow-up (4–10 months)	230	86.5	53	30.1	70	88.6	353	67.8

A total of 521 patients filled PROMs questionnaires at baseline, most of which were gathered by Romagna (51.0%), followed by Reggio-Emilia (33.8%) and Parma (15.2%). The percentage of patients with a valid follow-up (between 4 and 10 months from baseline) was similar between Romagna (86.5%) and Parma (88.6%), but considerably lower in Reggio-Emilia (30.1%), reflecting organizational differences in data collection processes.

## 4 Discussion

This study provides empirical evidence that the main barriers to effective use of health data in decentralized healthcare systems are organizational and governance-related, rather than technical.

Although a federated data infrastructure was successfully implemented across multiple healthcare organizations, substantial heterogeneity in data completeness and linkage was observed, reflecting differences in care pathways, data collection practices, and local governance structures.

These findings challenge the assumption that investments in digital infrastructure alone are sufficient to enable data-driven healthcare. Instead, they highlight the need to align health information systems with clinical workflows and service delivery models to ensure meaningful and comparable use of real-world data.

The report shows that a federated data linkage of administrative, clinical, and patient-reported data can be implemented at regional scale in the SSN. Beyond feasibility, our findings provide insight on how health information systems function in real-world settings, as well as the way in which organizational structures shape data availability and usability (21).

A critical observation resulting from this study is the marked heterogeneity in data completeness across participating centers. In particular, our findings indicate that the level of data completeness may be sensitive to both technical factors and local organizational practices. This variability should not be interpreted solely as a technical limitation or as a consequence of incomplete implementation.

In addition, there is a contribution, albeit minor, to be ascribed to an admixture of differences in clinical pathways, service organization, and data governance practices across healthcare management organization. In this sense, the information infrastructure realized in REWINDER becomes a mirror of the healthcare system itself, capturing not only patient and care delivery characteristics but also the way data access is governed.

The finding has important implications for the interpretation of population-level indicators. Differences in data availability may introduce systematic bias if not properly accounted for, particularly when comparing performance across providers or regions. Nevertheless, this variability offers an opportunity to identify organizational models associated with good practices in data capturing, data access and, potentially, with improved quality of care.

Data linkage poses additional challenges in large populations (such as people with NCDs), where all services delivered to an affected person should be linked over a lifetime, to ensure comprehensive coverage (10). This is borne out by the high-quality registries in the Nordic countries, where linked data have been systematically used for quality monitoring and research over single/multiple diseases (22). In Sweden, a collaborative effort allowed covering most of the population with diabetes, contributing to improving health outcomes (23). However, the lack of similar experiences in countries with large populations, raises the obvious question of an ideal scale to implement similar solutions (24).

The high proportion of missing data for key sociodemographic and behavioral variables in REWINDER highlights a broader structural limitation of current health information systems (25). Despite growing recognition of the role of social determinants in chronic disease outcomes, these dimensions are still poorly integrated into routine data collection. This shortcoming limits the ability to perform risk stratification for the purpose of designing targeted, equity-oriented interventions, and should therefore be considered as an important priority to tackle in future developments.

The integration of PROMs represents a further step toward person-centered care. International organizations, e.g., ICHOM, have rigorously identified global standard sets that can be systematically used for the scope of this work (8). However, their applicability is hampered by the limited coverage of clinical data and the practical difficulty of collecting PROMs at a granular level (13, 14).

In REWINDER, the collection of PROMs was feasible within routine clinical settings, confirming their potential value. However, the coverage achieved in this study remained limited and operational challenges were evident. In particular, the follow-up was heterogeneous, suggesting that the manual data collection approaches used in the project, may not be sustainable at population level. This raises the need of further studies and more advanced solutions to reach out to the target individuals.

### 4.1 Implications for scaling up at national and international level

The results obtained from local healthcare organizations as a basis for the construction of a federated regional register, lead to further reflections regarding the applicability of the model at national and international level.

In Italy, the geographical variability observed in healthcare within and across regions (26) has raised a debate on the relation between decentralization and fragmented health information (3).

In diabetes, the long-standing experience of the “Annali” produced by the Association of Medical Diabetologists (AMD) showed a continuous improvement of quality of care and outcomes obtained from specialized care (17). These results stimulated a debate on the opportunity of building a national diabetes register from regional experiences (1).

The federated approach of REWINDER represents a potentially viable solution to exploit the intrinsic characteristics of decentralization as an advantage, rather than an obstacle, in this endeavor. There are indeed favorable conditions to adopt this model.

In the SSN, each citizen is assigned to a unique territorial area, healthcare organization and general practitioner. People with major chronic diseases are registered in an exemption list, allowing free access to all relevant services.

Consequent to governing mechanisms at different levels (27), and the need to create common standards, the SSN has nurtured a rich information infrastructure allowing data linkage and comparability between the local and central levels, using standardized definitions and a reliable UPI managed by the Ministry of Finance for the continuous control of health expenditure (7).

In this framework, REWINDER outlined a scalable model for national data collection, in which:

- the extraction of longitudinal cohorts can be facilitated by validated regional algorithms applied on top of administrative data in each region;
- the integration of software used in diabetes clinics with standardized administrative data across the country may support data extraction and linkage using a common pseudonym;
- the collection and data linkage of PROMs for psychometric profiling (28, 29) is possible through personal consent. However, secure automated means are needed for sustainable data collection, avoiding hardcopy questionnaires, email reminders, or custom-made forms using Redcap (30);
- use of open-source software (OSS) can facilitate the process. However, its adoption should be further encouraged and authorized, consistently with EU recommendations (31).

Scaling up at international level may be possible through the implementation of the EHDS regulation and specific programs such as JACARDI, which promise to leverage interoperable and sustainable policy-relevant data infrastructures for NCDs (10).

In this broader context, REWINDER can offer further scope to overcome the fragmentation of silos, building on local collaboration to enhance comparability, while ensuring conformity with privacy legislation (32–34).

Overall, our findings show the broad range of opportunities and challenges associated with the establishment of federated health data infrastructures.

The following implications should be considered at the European level:

- local healthcare organizations can perform complex data processing, but need to avoid bottlenecks and long operating times through specialized software and outsourcing services for pseudonymization, data linkage and Extract, Transform, and Load (ETL) operations;
- highly informative clinical data can be directly available from outpatient clinics, but coverage of the target population may depend from medical practices. Bias can be reduced and completeness improved by integrating other sources, e.g., laboratory results;
- access and manipulation of clinical databases may be restricted by contractual agreements, whose adherence to EU regulations must be checked to ensure legal and technical viability;
- routine collection of PROMs is feasible, but should be tuned to realistic timelines and sample size targets. The list of subjects included in cohorts must be available prior to enrolling patients, to ensure synchronized follow-up with daily visits, using computerized reminders;
- the variability of practices of data governance may be reduced through the use of EU-approved checklists for the implementation of federated networks by local healthcare organizations. The conditions for use of research-oriented OSS should be clarified to ensure its wide adoption.

Considering the above recommendations may help building modern tools that can associate the production of NCD indicators to the provision of person-centered care, e.g., smartphone apps integrated with population-based databases (35).

These developments can benefit from the long standing experience of registry networks, e.g., EUBIROD (12, 21, 36), whose members are contributing to the “Collaborative Health Information European Framework” (CHIEF) coordinated by the European Commission’s Joint Research Centre (JRC) (37–40). The recommendations emerging from CHIEF may help evaluating the relevance of REWINDER for the construction of a pan-European diabetes register (41).

### 4.2 Strengths and limitations

This study is based on a large population-based cohort and integrates multiple data sources within a federated infrastructure, demonstrating feasibility of sub-national registries that can be managed by local healthcare organizations in a real-world setting.

A common technological platform eliminates major sources of bias induced by heterogeneous approaches of data extraction, transformation and analysis, allowing to focus on variations due to real-world care practices. In this way, the federated network operates as an epidemiologic equalizer, allowing fair comparisons of data quality measures, e.g., patterns of missing data and failed linkages, as proxies of quality of care monitoring ability. A federated regional system may highlight potential gaps and organizational improvements to enhance the continuity of care for people with chronic diseases in the community.

However, several limitations need to be also acknowledged.

Firstly, heterogeneity in data completeness across centers may limit comparability. The study included only three geographical areas and the results may not necessarily apply to other geographical areas and/or represent the variety of social and cultural characteristics in all decentralized contexts.

Secondly, high levels of missing clinical and socio-demographic data constrain the assessment of relevant determinants for population-based investigation.

Thirdly, we did not tackle the complex issues of semantic interoperability such as the adaptation to FAIR principles (41), the consideration of which may improve data integration and population-based indicators, both particularly relevant for the rapid evolution of EU health-data regulations.

Finally, the implementation of PROMs was limited in scale and may not be sustainable without automation.

The above limitations reflect structural characteristics of current health information systems rather than study-specific weaknesses.

## 5 Conclusion

REWINDER provides a scalable and policy-relevant model for federated health data infrastructures in decentralized healthcare systems. The study may be used as a model for the collaborative construction of federated registers, where healthcare organizations can provide the essential levels of health information (42).

Our findings suggest that solid information infrastructure require not only technical solutions, but coordinated organizational change across healthcare systems. Addressing data completeness, governance, and operational processes in the context of the European Health Data Space will promote wider adoption and maximize the impact of real-world data for chronic disease management in Europe.

## Data Availability

The datasets presented in this article are not readily available due to legal and ethical restrictions. Analyses were conducted within a federated infrastructure, where individual-level data remain locally stored and only aggregated results are accessible. Requests to access the datasets should be directed to paolo.dibartolo@auslromagna.it.

## Footnotes

1
https://ngsp.org/ifcc.asp
