# Supplementary material for: Implementing a federated regional diabetes register in a decentralized health system: implications for healthcare organization and the European Health Data Space
Source: Front Public Health. 2026 Jul 16;14:1864673. doi: 10.3389/fpubh.2026.1864673 (PMC13422561; doi:10.3389/fpubh.2026.1864673)
Supplement: Supplementary file 1 [file Data_Sheet_1.pdf]

SUPPLEMENTARY MATERIALS

Description of REWINDER databases

The administrative databases implemented in REWINDER include the following:

- personal demographic data (**Table S-L1**)
- hospital discharge abstracts (**Tables S-L2 to S-L4**)
- admissions to the emergency departments (**Table S-L5**)
- pharmaceutical prescriptions (**Table S-L6**)
- information on COVID-19 (**Table S-L7**).

Repeated measurements were collapsed into annual summaries for statistical analysis (see **Tables S-R1 to S-R6** in supplementary data).

All centres used the same MetaClinic system to collect baseline data and repeated visits (see **Tables S-L8A/B**).

PROMs have been included in **Table S-L9**.

Table S-L1. REW_ANAG (Source: SDO, ARA; all people with diabetes in the study cohort)

CATEGORY	Sub-category	Variable ID	ITEM	TYPE	RESPONSE OPTIONS
ID	ID	**ID_PAZIENTE**	Tax file number (ENCRYPTED)	SHA-256	
Case-mix variables	Demographic Factors	SESSO	Sex	Numeric	1 = Male; 2 = Female; 3 = Other; 999 = Undisclosed
		ANNO_NASCITA	Year of Birth	Numeric	YYYY
	Diagnosis Profile	ANNO_DIAGNOSI	Year of Diagnosis	Numeric	Year of Diagnosis
	Diagnosis Source	FONTE	Source	Character	SDO=Hospital Discharges; FAR=Prescriptions; ESE=Exemptions
Outcomes	Survival	ANNO_DECESSO	Year of Death	Numeric	YYYY

Table S-L2. REW_HOSP_DPL (Source: SDO; all hospital admissions for people with diabetes, 2019-2024)

CATEGORY	Sub-category	Variable ID	ITEM	TYPE	RESPONSE OPTIONS
ID	ID	**ID_PAZIENTE**	Tax file number (ENCRYPTED)	SHA-256	
		**COD_EVENTO**	Unique Code of Hospital Admission	Character	
Healthcare Outcomes	Healthcare utilization	DAT_RICOVERO	Admission date	Date	YYYY-MM-DD
		DAT_DIMISSIONE	Discharge date	Date	YYYY-MM-DD
		COD_MODALITA_DIM	Discharge mode	Numeric	
		COD_TIPO_COVID	Covid diagnosis	Numeric	

Table S-L3. REW_HOSP_DIAG_DPL (Source: SDO; all hospital admissions for people with diabetes, 2019-2024)

CATEGORY	Sub-category	Variable ID	ITEM	TYPE	RESPONSE OPTIONS
ID	ID	**COD_EVENTO**	Unique Code of Hospital Admission	Character	
Healthcare Outcomes	Healthcare utilization	COD_DIAGNOSI	Diagnosis Code	Character	XXX.XX
		FLG_DIAGNOSI_PRI	Flag Principal Diagnosis	Character	XXX.XX

Table S-L4. REW_HOSP_INT_DPL (Source: SDO; all hospital admissions for people with diabetes, 2019-2024)

CATEGORY	Sub-category	Variable ID	ITEM	TYPE	RESPONSE OPTIONS
ID	ID	**COD_EVENTO**	Unique Code of Hospital Admission		
Healthcare outcomes	Healthcare utilization	COD_INTERVENTO	Code of Intervention	Numeric	XXX.XX
		DAT_INTERVENTO	Intervention date	Date	YYYY-MM-DD
		FLG_INTERVENTO_PRI	Flag principal intervention	Binary	0/1

Table S-L5. REW_EMUR_DPL (Source: EMUR; access to emergency services for all people with diabetes in the study cohort)

CATEGORY	Sub-category	Variable ID	ITEM	TYPE	RESPONSE OPTIONS
ID	ID	**ID_PAZIENTE**	Tax file number (ENCRYPTED)	SHA-256	
Healthcare outcomes	Healthcare utilization	**DAT_ARRIVO**	Access date	Date	YYYY-MM-DD
		COD_DIAGNOSI_PRI	Principal diagnosis code	Character	XXX.XX
		N_ACCESSI	Number of accesses	Number of accesses	N

Table S-L6. REW_PHARM_DPL (Source: AFT/FED Pharmaceutical prescriptions, pharmacies, hospital distribution, people with diabetes 2019-2024)

CATEGORY	Sub-category	Variable ID	ITEM	TYPE	RESPONSE OPTIONS
ID	ID	**ID_PAZIENTE**	Tax file number (ENCRYPTED)	SHA-256	
Healthcare outcomes	Healthcare utilization	**DAT_EROGAZIONE**	Prescription date	Date	YYYY-MM-DDi
		COD_ATC4	ATC Code	Numeric	XXX
		QTA_CONF	Number of packets	Numeric	X
		DDD	Daily doses	Numeric	XXX

Table S-L7. REW_COVID (COVID-19 infections, all people with diabetes 2020-2024); Timing: Annually

CATEGORY	Sub-category	Variable ID	ITEM	TYPE	RESPONSE OPTIONS
ID	ID	**ID_PAZIENTE**	Tax file number (ENCRYPTED)	SHA-256	
Covid	Covid	**YEAR**	Reference Year	Numeric	YYYY
		COVID	Covid infection	Numeric	Number of Covid infections
		COVID_IMMU	Covid immunization	Numeric	Number of Covid Immunizations
		COVID_IMMU_TYPE	Covid Vaccine	Text list	Type of Covid vaccine
		COVID_HOSP_MED	Covid Hospitalization non Intensive	Numeric	Number of days hospitalized
		COVID_HOSP_IC	Covid Hospitalization Intensive	Numeric	Number of days hospitalized in Intensive care

Table S-L8A. CLIN_PERSONS

CATEGORY	Sub-category	Variable ID	ITEM	TYPE	RESPONSE OPTIONS
ID	ID	**ID_PAZIENTE**	Tax file number (ENCRYPTED)	SHA-256	
		**CENTRE_ID**	Provider Centre Code	Character	7 characters
		SEX	Sex	Numeric	1 = Male; 2 = Female; 3 = Other; 999 = Undisclosed
		YOB	Year of Birth	Numeric	YYYY
		YEAR_DIAG	Year of Diagnosis	Numeric	Year of Diagnosis
		DATE_1ST_ACCESS	Date of First Access	Character	YYYY-MM-DD
		DM_TYPE	Diabetes mellitus subtype	Categorical	0 = No diabetes; 1 = Diabetes mellitus type 1; 2 = Diabetes mellitus type 2; 999 = Unknown
		ATTIVO_1719	Flag for patient activity status between 2017-2019	Binary	0 = Inactive, 1 = Active

Table S-L8B. CLIN_EVENTS

CATEGORY	Sub-category	Variable ID	ITEM	TYPE	RESPONSE OPTIONS
ID	ID	**ID_PAZIENTE**	Tax file number (ENCRYPTED)	SHA-256	
Time	Time	**DAT_VISITA**	Visit date	Date	YYYY-MM-DD
Case-mix variables Demographic factors	Demographic Factors	EDU_LEVEL	Level of education	Categorical	0= None; 1= Primary; 2= Secondary; 3= Tertiary
Case-mix variables Diagnosis Profile	Diagnosis Profile	DM_TYPE	Diabetes mellitus subtype	Categorical	0 = No diabetes; 1 = Diabetes mellitus type 1; 2 = Diabetes mellitus type 2; 999 = Unknown
		YEAR_DIAG	Year of Diagnosis	Numeric	YYYY
	Baseline health status	CVD_COMORB	Cardiovascular Comorbidities	Multiple answer	0 = no other diseases; 1 = Heart Disease (Angina, heart attack, or HF); 2 = High Blood Pressure; 3 = Atrial Fibrillation or Flutter; 4 = Chronic Pulmonary Disease (Asthma, Chronic Bronchitis, COPD, Emphysema); 5 = Diabetes; 6 = Peripheral Artery Disease; 7 = Myocardial Infarction; 8 = Obesity; 9 = Stroke; 10 = Ulcer or stomach disease; 11 = Renal Insufficiency; 12 = Liver Disease; 13 = Anemia or other blood disease; 14 = Cancer/Other Cancer in last 5 years; 15 = AIDS/Immunodeficiency; 16 = Presence/History of Depression; 17 = Anxiety or Neuroses; 18 = Presence/History of Psychotic Mental Illness (e.g., Schizophrenia); 19 = Substance Abuse; 20 = Osteoarthritis, degenerative arthritis; 21 = Rheumatoid Arthritis; 22 = Periodontal Disease; 888 = Other

CATEGORY	Sub-category	Variable ID	ITEM	TYPE	RESPONSE OPTIONS
					Medical Problems
Case-mix variables Lifestyle and Social Factors	Lifestyle and Social Factors	SMOK_STAT	Smoking status	Categorical	0 = Current every day smoker; 1 = Current weekly smoker; 2 = Former smoker; 3 = Never smoker; 4 = Others; 999 = Unknown if ever smoked
		ALC_FREQ1	Alcohol Frequency	Categorical	0= Every day/7 days per week; 1= 5 to 6 days per week; 2= 3 to 4 days per week; 3= 1 to 2 days per week; 4= 1 to 3 days per month; 5= Never
		ALC_OCCAS	Alcohol Amount per drinking occasion	Numeric	Average number of units consumed in whole numbers
		PHYS_ACT	Physical Activity	Categorical	0 = No; 1 = Yes; 999 = Unknown
Clinician-reported Outcomes Diabetes Control	Glycemic Control	HBA1C	Glycemic Control HbA1c	Numeric	Numerical value of HbA1c
		HBA1C_UNIT	Glycemic Control<br>Units of HbA1c	Categorical	0= mmol/mol; 1= %
		TIR	Glycemic Control Time in range	Numeric	Numerical value of time in range
Clinician-reported Outcomes Diabetes Control	Intermediate outcomes	SBP	Systolic blood pressure	Numeric	Numerical systolic BP in mmHG
		DBP	Diastolic blood pressure	Numeric	Numerical diastolic BP in mmHg
		BL_GLUC	Blood Glucose	Numeric	Value of Blood Glucose
		BL_GLUC_UN	Blood Glucose Unit of Measure	Categorical	0= mmol/mol; 1= %
		LIP_TCHOL	Lipid Profile: Total Cholesterol	Numeric	Numerical value of blood total cholesterol concentration
		LIP_TCHOL_UNI	Units of Total Cholesterol	Categorical	0= mmol/mol; 1= %
		LIP_LDL	Lipid Profile: LDL Cholesterol	Numeric	Numerical value of blood LDL cholesterol concentration
		LIP_LDL_UNI	Units of LDL Cholesterol	Categorical	0= mmol/mol; 1= %
		LIP_HDL	Lipid Profile: HDL Cholesterol	Numeric	Numerical value of blood HDL cholesterol concentration
		LIP_HDL_UNI	Units of HDL Cholesterol	Categorical	0= mmol/mol; 1= %
		LIP_TRI	Lipid Profile: Triglycerides	Numeric	Numerical value of blood triglyceride concentration
		LIP_TRI_UNI	Units of Triglycerides	Categorical	0= mmol/mol; 1= %
		WGT_VAL	Body weight	Numeric	Numerical value of weight
		WG_UNIT	Body weight units	Categorical	1 = kilograms; 2 = lbs
		HGT_VAL_DIA	Body height	Numeric	Numerical value of height
		HGT_UNIT	Body height units	Categorical	1 = centimeters; 2 = inches
		WAISTC	Waist Circumference	Numeric	Numerical value of waist circumference in centimeters
	Hypoglycemia	HYPOL3	Hypoglycemia - Level 3	Categorical	0= 0; 1= 1; 2= 2; 3= more than 2; 999= Unknown
		HYPOL2	Hypoglycemia - Level 2	Numeric	Numeric Response
		HYPOL2_AWAR	Hypoglycemia - Level 2<br>Loss of awareness of hypoglycemia	Categorical	0 = No; 1 = Yes; 999 = Unknown
	Micro- and macrovascular	MI	Myocardial infarction	Categorical	0 = No; 1 = Yes; 999 = Unknown
		CVD	Cerebrovascular Disease - Acute events	Categorical	0 = No; 1 = Yes; 999 = Unknown

CATEGORY	Sub-category	Variable ID	ITEM	TYPE	RESPONSE OPTIONS
	complications	LL_AMP	Lower Limb Amputation	Categorical	0 = No; 1 = Yes; 999 = Unknown
		LL_AMP_LEV	Lower Limb Amputation Level	Categorical	0= Distal to the ankle joint; 1= Below knee; 2= Above knee; 999= Unknown
		VIS_THREAT	Visual Outcomes Diabetes-related Sight Threatening Conditions	Multiple answer	0= Non-proliferative diabetic retinopathy; 1= Proliferative diabetic retinopathy; 2= Unspecified diabetic retinopathy; 3= Macular edema; 4= Other; 5= No sight threatening conditions; 999= Unknown
		AUT_NEU	Autonomic Neuropathy	Categorical	0 = No; 1 = Yes; 999 = Unknown
		PERI_NEU_CLI	Peripheral Neuropathy - Clinician Diagnosis	Categorical	0 = No; 1 = Yes; 999 = Unknown
		PERI_NEU_PAT	Peripheral Neuropathy - Symptoms experienced by person with diabetes	Categorical	0= Numbness; 1= Pain; 2= Paresthesia; 3= Asymptomatic/ No symptoms; 999= Unknown
		CHARC_F	Charcot's Foot	Categorical	0 = No; 1 = Yes; 999 = Unknown
		LL_ULC	Lower Limb Ulcers Active lower limb ulcer present?	Categorical	0 = No; 1 = Yes; 999 = Unknown
		PAD	Peripheral artery disease	Categorical	0 = No; 1 = Yes; 999 = Unknown
		PAD_PAT	Peripheral artery disease - Symptoms experienced by person with diabetes	Categorical	0= No; 1= Yes, intermittent claudication; 2= Yes, rest pain; 999= Unknown
		IHD	Ischemic Heart Disease	Categorical	0 = No; 1 = Yes; 999 = Unknown; 999 = Unknown
		HF	Heart failure	Categorical	0 = No; 1 = Yes; 999 = Unknown
		CHRSTAGE	Chronic Heart Failure Staging	Categorical	0= Stage A (At high risk of HF without structural heart disease or symptoms of HF); 1= Stage B (Structural heart disease but without signs or symptoms of HF); 2= Stage C (Structural heart disease with prior or current symptoms of HF); 3= Stage D (Refractory HF requiring specialist interventions); 4 = Stage unknown; 999= Not assessed
		EGFR	Estimated glomerular filtration rate	Numeric	None
		RFTACR	Renal Function Tests/Moderate to Severe Kidney Disease ACR	Categorical	0= ACR <30mg/g or <3mg/mmol; 1= ACR 30-300mg/g or 3-30mg/mmol; 2= ACR > 300 mg/g or >30mg/mmol; 3= ACR unknown
		DIAL_DEP	Dialysis dependent	Categorical	0 = No; 1 = Yes; 999 = Unknown
		CD	Cerebrovascular disease	Categorical	0 = No; 1 = Yes; 999 = Unknown
		SEX_DYSF	Sexual Dysfunction	Categorical	0 = No; 1 = Yes; 999 = Unknown
		ATTIVO_1719	Flag for patient activity status between 2017-2019	Binary	0 = Inactive, 1 = Active

Table S-L9. PROMS

CATEGORY	Sub-category	Variable ID	ITEM	TYPE	RESPONSE OPTIONS
ID	ID	**ID_PAZIENTE**	Tax file number (ENCRYPTED)	SHA-256	
Date	Recording date	**DATE**	Date		YYYY-MM-DD
Patient-reported outcomes	Psychological Wellbeing (WHO-5 Well-Being Index, www.who-5.org)	WHO-5_Q01	Question 1	Ordinal	5 = All time; 4 = Most; 3 = More than half 2 = Less than half; 1 = Some time; 0 = no time
		WHO-5_Q02	Question 2	Ordinal	5 = All time; 4 = Most; 3 = More than half 2 = Less than half; 1 = Some time; 0 = no time
		WHO-5_Q03	Question 3	Ordinal	5 = All time; 4 = Most; 3 = More than half 2 = Less than half; 1 = Some time; 0 = no time
		WHO-5_Q04	Question 4	Ordinal	5 = All time; 4 = Most; 3 = More than half 2 = Less than half; 1 = Some time; 0 = no time
		WHO-5_Q05	Question 5	Ordinal	5 = All time; 4 = Most; 3 = More than half 2 = Less than half; 1 = Some time; 0 = no time
	Health-related Quality of Life/Self-Reported Health Status (PAID Diabetes Distress Score, http://www.joslin.org)	PAID_Q1	Question 1	Ordinal	0 = No problem; 1 = Minor; 2 = Moderate; 3 = Somewhat serious; 4 = Serious
		PAID_Q2	Question 2	Ordinal	0 = No problem; 1 = Minor; 2 = Moderate; 3 = Somewhat serious; 4 = Serious
		PAID_Q3	Question 3	Ordinal	0 = No problem; 1 = Minor; 2 = Moderate; 3 = Somewhat serious; 4 = Serious
		PAID_Q4	Question 4	Ordinal	0 = No problem; 1 = Minor; 2 = Moderate; 3 = Somewhat serious; 4 = Serious
		PAID_Q5	Question 5	Ordinal	0 = No problem; 1 = Minor; 2 = Moderate; 3 = Somewhat serious; 4 = Serious
	Depression (PHQ_9 Depression score, https://www.phqscreeners.com)	PHQ_9_Q1a	Question 1a	Ordinal	0 = Not at all; 1 = Several days; 2 = More than half the days; 3 = Nearly every day
		PHQ_9_Q1b	Question 1b	Ordinal	0 = Not at all; 1 = Several days; 2 = More than half the days; 3 = Nearly every day
		PHQ_9_Q1c	Question 1c	Ordinal	0 = Not at all; 1 = Several days; 2 = More than half the days; 3 = Nearly every day
		PHQ_9_Q1d	Question 1d	Ordinal	0 = Not at all; 1 = Several days; 2 = More than half the days; 3 = Nearly every day
		PHQ_9_Q1e	Question 1e	Ordinal	0 = Not at all; 1 = Several days; 2 = More than half the days; 3 = Nearly every day
		PHQ_9_Q1f	Question 1f	Ordinal	0 = Not at all; 1 = Several days; 2 = More than half the days; 3 = Nearly every day
		PHQ_9_Q1g	Question 1g	Ordinal	0 = Not at all; 1 = Several days; 2 = More than half the days; 3 = Nearly every day
		PHQ_9_Q1h	Question 1h	Ordinal	0 = Not at all; 1 = Several days; 2 = More than half the days; 3 = Nearly every day
		PHQ_9_Q1i	Question 1i	Ordinal	0 = Not at all; 1 = Several days; 2 = More than half the days; 3 = Nearly every day
		PHQ_9_Q2	Question 2	Ordinal	0 = Not difficult; 1 = Somewhat difficult; 2 = Very; 3 = Extremely, 0 = No problem; 1 = Minor; 2 = Moderate; 3 = Somewhat serious; 4 = Serious

Table S-R1. ANAG

CATEGORY	Sub-category	Variable ID	ITEM	TYPE	RESPONSE OPTIONS
ID	ID	**ID_PAZIENTE**	Tax file number (ENCRYPTED)	SHA-256	
Case-mix variables Demographic factors	Demographic Factors	SESSO	Sex	Categorical	1 = Male; 0 = Female
		ANNO_NASCITA	Year of Birth	Numerical	YYYY
Case-mix variables Diagnosis Profile	Diagnosis Profile	ANNO_DIAGNOSI	Year of Diagnosis	Numerical	YYYY
Clinician-reported Outcomes Survival	Survival	VITAL_STATUS	Vital Status	Binary	0 = No; 1 = Yes
		ANNO_DECESSO	Year of death	Numerical	YYYY

Table S-R4. EMUR (Timing: Annually)

CATEGORY	Sub-category	Variable ID	ITEM	TYPE	RESPONSE OPTIONS
ID	ID	**ID_PAZIENTE**	Tax file number (ENCRYPTED)	SHA-256	
		**YEAR**	Reference Year	Numeric	YYYY
		ERUTIL	Emergency Room Utilization	Numeric	N of emergency room attendances (000 if unknown)

Table S-R5. COVID (Timing: Annually)

CATEGORY	Sub-category	Variable ID	ITEM	TYPE	RESPONSE OPTIONS
ID	ID	**ID_PAZIENTE**	Tax file number (ENCRYPTED)	SHA-256	
Covid	Covid	**YEAR**	Reference Year	Numeric	YYYY
		COVID	Covid infection	Numeric	Number of Covid infections
		COVID_Immu	Covid immunization	Numeric	Number of Covid Immunizations
		COVID_Immu_Type	Covid Vaccine	Text list	Type of Covid vaccine
		COVID_Hosp_Med	Covid Hospitalization non Intensive	Numeric	Number of days hospitalized
		COVID_Hosp_IC	Covid Hospitalization Intensive	Numeric	Number of days hospitalized in Intensive care

Table S-R2. HOSP (Timing: Annually)

CATEGORY	Sub-category	Variable ID	ITEM	TYPE	RESPONSE OPTIONS
ID	ID	**ID_PAZIENTE**	Tax file number (ENCRYPTED)	SHA-256	
		**YEAR**	Reference Year		
Case-mix variables Diagnosis Profile	Comorbidities	CVD_COMORB	Cardiovascular Comorbidities	Binary	0 = No; 1 = Yes
		CVD_COMORB_HEART	Heart Disease (Angina, heart attack, or HF)	Binary	0 = No; 1 = Yes
		CVD_COMORB_BP	High Blood Pressure	Binary	0 = No; 1 = Yes
		CVD_COMORB_AF	Atrial Fibrillation or Flutter	Binary	0 = No; 1 = Yes
		CVD_COMORB_COPD	Chronic Pulmonary Disease (Asthma, Chronic Bronchitis, COPD, Emphysema)	Binary	0 = No; 1 = Yes
		CVD_COMORB_DIAB	Diabetes	Binary	0 = No; 1 = Yes
		CVD_COMORB_PAD	Peripheral Artery Disease	Binary	0 = No; 1 = Yes
		CVD_COMORB_MI	Myocardial Infarction	Binary	0 = No; 1 = Yes
		CVD_COMORB_OBE	Obesity	Binary	0 = No; 1 = Yes
		CVD_COMORB_STRO	Stroke	Binary	0 = No; 1 = Yes
		CVD_COMORB_ULC	Ulcer or stomach disease	Binary	0 = No; 1 = Yes
		CVD_COMORB_REN	Renal Insufficiency	Binary	0 = No; 1 = Yes
		CVD_COMORB_LIV	Liver Disease	Binary	0 = No; 1 = Yes
		CVD_COMORB_ANE	Anemia or other blood disease	Binary	0 = No; 1 = Yes
		CVD_COMORB_CANC	Cancer/Other Cancer in last 5 years	Binary	0 = No; 1 = Yes
		CVD_COMORB_AIDS	AIDS/Immunodeficiency	Binary	0 = No; 1 = Yes
		CVD_COMORB_DEP	Presence/History of Depression	Binary	0 = No; 1 = Yes
		CVD_COMORB_ANX	Anxiety or Neuroses	Binary	0 = No; 1 = Yes
		CVD_COMORB_PMH	Presence/History of Psychotic Mental Illness (e.g. Schizophrenia)	Binary	0 = No; 1 = Yes
		CVD_COMORB_ABU	Substance Abuse	Binary	0 = No; 1 = Yes
		CVD_COMORB_OST	Osteoarthritis, degenerative arthritis	Binary	0 = No; 1 = Yes
		CVD_COMORB_RHA	Rheumatoid Arthritis	Binary	0 = No; 1 = Yes
		CVD_COMORB_PD	Periodontal Disease	Binary	0 = No; 1 = Yes
		CVD_COMORB_OTH	Other Medical Problems	Binary	0 = No; 1 = Yes
Case-mix variables Procedures Profile	Cardiovascular Procedures	CVD_PROC_TR	Cardiovascular Procedural Treatment	Binary	0 = No; 1 = Yes
		CVD_PROC_TR_CABG	CABG	Binary	0 = No; 1 = Yes
		CVD_PROC_TR_VALVE	Valve surgery	Binary	0 = No; 1 = Yes
		CVD_PROC_TR_ANY	Any cardiac surgery	Binary	0 = No; 1 = Yes
		CVD_PROC_TR_P_PCP	Prior percutaneous coronary procedure	Binary	0 = No; 1 = Yes
		CVD_PROC_TR_P_PVC	Prior percutaneous valve procedure	Binary	0 = No; 1 = Yes
		CVD_PROC_TR_O_PI	Another; percutaneous intervention (e.g. catheter ablation)	Binary	0 = No; 1 = Yes

CATEGORY	Sub-category	Variable ID	ITEM	TYPE	RESPONSE OPTIONS
	Ketoacidosis	DKAHHS	Diabetic Ketoacidosis and Hyperosmolar Hyperglycemic Syndrome	Categorical	0= No; 1=Yes, only DKA; 2= Yes, only HHS; 3= Yes, both
	Hypoglycemia	HYPOL3	Hypoglycemia - Level 3	Categorical	0 = 0; 1= 1; 2= 2; 3=>2
		HYPOL2	Hypoglycemia - Level 2	Numeric	Numeric Response
		HYPOL2_AWAR	Hypoglycemia - Level 2 – Loss of awareness	Binary	0 = No; 1 = Yes
	Acute CVD	MI	Myocardial infarction	Binary	0 = No; 1 = Yes
		CVD	Cerebrovascular Disease - Acute events	Binary	0 = No; 1 = Yes
	Micro- and macrovascular complications	LL_AMP	Lower Limb Amputation	Binary	0 = No; 1 = Yes
		LL_AMP_LEV	Lower Limb Amputation Level	Categorical	0= None; 1=Distal to the ankle joint; 1= Below knee; 2= Above knee;
		VIS_THREAT	Visual Outcomes - Diabetes-related Sight Threatening Conditions	Binary	0 = No; 1 = Yes
		VIS_MACULA	Visual Outcomes - Macular edema	Binary	0 = No; 1 = Yes
		VIS_RETINO	Visual Outcomes – Diabetes retinopathy	Categorical	0 = None; 1=Non-proliferative; 2= Proliferative; 3= Unspecified
		AUT_NEU	Autonomic Neuropathy	Binary	0 = No; 1 = Yes
		PERI_NEU_CLI	Peripheral Neuropathy - Clinician Diagnosis	Binary	0 = No; 1 = Yes
		PERI_NEU_PAT	Peripheral Neuropathy - Symptoms experienced by person with diabetes	Categorical	0= None; 1= Numbness; 2= Pain; 3= Paresthesia
		CHARC_F	Charcot's Foot	Binary	0 = No; 1 = Yes
		LL_ULC	Lower Limb Ulcers/Active	Binary	0 = No; 1 = Yes
		PAD	Peripheral artery disease	Binary	0 = No; 1 = Yes
		IHD	Ischemic Heart Disease	Binary	0 = No; 1 = Yes
		HF	Heart failure	Binary	0 = No; 1 = Yes
		DIAL_DEP	Dialysis dependent	Binary	0 = No; 1 = Yes
		CD	Cerebrovascular disease	Binary	0 = No; 1 = Yes
Clinician-reported Outcomes Health Services	Healthcare utilization	HOSPADM_N	Number of Hospitalizations	Numeric	
		HOSPADMIN_NLOS	Total Number of Hospitalization Days	Numeric	
	Cardiovascular Diagnoses	CVD_DIAG_ANY	Cardiovascular diagnoses: Any	Binary	0 = No; 1 = Yes
		CVD_DIAG_AKD	Cardiovascular diagnoses: Acute Kidney Injury	Binary	0 = No; 1 = Yes
		CVD_DIAG_FOOT	Cardiovascular diagnoses: Foot- and lower limb-related complications	Binary	0 = No; 1 = Yes
		CVD_DIAG_META	Cardiovascular diagnoses: Acute metabolic complications	Binary	0 = No; 1 = Yes
		CVD_DIAG_ACD	Cardiovascular diagnoses: Acute cardiovascular diagnoses	Binary	0 = No; 1 = Yes

Table S-R3. PHARM (Timing: Annually)

CATEGORY	Sub-category	Variable ID	ITEM	TYPE	RESPONSE OPTIONS
**ID**	ID	**ID_PAZIENTE**	Tax file number (ENCRYPTED)	SHA-256	
Clinician-reported Outcomes Health Services	Healthcare utilization	CVD_TX	Cardiovascular Pharmacological Treatment	Binary	0 = No; 1 = Yes
		CVD_TX_INS	Insulin therapy	Binary	0 = No; 1 = Yes
		CVD_TX_STAT	Statins (e.g. atorvastatin,rosuvastatin, etc.)	Binary	0 = No; 1 = Yes
		CVD_TX_SGLT2	SGLT2 Inhibitors	Binary	0 = No; 1 = Yes
		CVD_TX_MET	Metformin	Binary	0 = No; 1 = Yes
		CVD_TX_SULF	Sulfonylurea	Binary	0 = No; 1 = Yes
		CVD_TX_GLP1	GLP1 Agonists	Binary	0 = No; 1 = Yes
		CVD_TX_ANTI	Antilplatelet agents (e.g. ASA, Clopidogrel, Prasugrel, Ticagrelor, etc.)	Binary	0 = No; 1 = Yes
		CVD_TX_NOA	Novel oral anticoagulant (NOAC) (e.g. rivaroxaban, dabigatran, etc.)	Binary	0 = No; 1 = Yes
		CVD_TX_ISO	Isosorbide Dinitrate	Binary	0 = No; 1 = Yes
		CVD_TX_ANG	Angiotensin Receptor Blocker/Neprilysin Inhibitor Combination	Binary	0 = No; 1 = Yes
		CVD_TX_BB	Beta-blockers (e.g. metoprolol, bisoprolol, propranolol, etc.)	Binary	0 = No; 1 = Yes
		CVD_TX_VITK	Vitamin K Antagonists (e.g. warfarin)	Binary	0 = No; 1 = Yes
		CVD_TX_MIN	Mineralocorticoid Receptor Antagonists (e.g spironolactone)	Binary	0 = No; 1 = Yes
		CVD_TX_ANG2	Angiotensin II Receptor Antagonist (e.g. losartan, irbesartan, candesartan, etc.)	Binary	0 = No; 1 = Yes
		CVD_TX_ACE	ACE Inhibitors (e.g. enalapril, captopril, etc.)	Binary	0 = No; 1 = Yes
		CVD_TX_LD	Loop diuretics (e.g. furosemide)	Binary	0 = No; 1 = Yes
		CVD_TX_OTH	Other	Binary	0 = No; 1 = Yes

Table S-R6. CLIN (Conditions – any recorded during the year; Measurements - last recorded during the year)

CATEGORY	Sub-category	Variable ID	ITEM	TYPE	RESPONSE OPTIONS
ID	ID	**ID_PAZIENTE**	Tax file number (ENCRYPTED)	SHA-256	
Time	Time	**YEAR**	Reference Year	Year	YYYY
Case-mix variables	Demographic Factors	EDU_LEVEL	Level of education	Categorical	0= None; 1= Primary; 2= Secondary; 3= Tertiary
	Diabetes Diagnosis	DM_TYPE	Diabetes mellitus subtype	Categorical	0 = None; 1 = Type 1; 2 = Type 2; 3 = Other
		YEAR_DIAG	Year of Diagnosis	Numeric	XXXX
	Comorbidities	CVD_COMORB	Cardiovascular Comorbidities	Binary	0 = No; 1 = Yes
		CVD_COMORB_HEART	Heart Disease (Angina, heart attack, or HF)	Binary	0 = No; 1 = Yes
		CVD_COMORB_BP	High Blood Pressure	Binary	0 = No; 1 = Yes
		CVD_COMORB_AF	Atrial Fibrillation or Flutter	Binary	0 = No; 1 = Yes
		CVD_COMORB_COPD	Chronic Pulmonary Disease (Asthma, Chronic Bronchitis, COPD, Emphysema)	Binary	0 = No; 1 = Yes
		CVD_COMORB_DIAB	Diabetes	Binary	0 = No; 1 = Yes
		CVD_COMORB_PAD	Peripheral Artery Disease	Binary	0 = No; 1 = Yes
		CVD_COMORB_MI	Myocardial Infarction	Binary	0 = No; 1 = Yes
		CVD_COMORB_OBE	Obesity	Binary	0 = No; 1 = Yes
		CVD_COMORB_STRO	Stroke	Binary	0 = No; 1 = Yes
		CVD_COMORB_ULC	Ulcer or stomach disease	Binary	0 = No; 1 = Yes
		CVD_COMORB_REN	Renal Insufficiency	Binary	0 = No; 1 = Yes
		CVD_COMORB_LIV	Liver Disease	Binary	0 = No; 1 = Yes
		CVD_COMORB_ANE	Anemia or other blood disease	Binary	0 = No; 1 = Yes
		CVD_COMORB_CANC	Cancer/Other Cancer in last 5 years	Binary	0 = No; 1 = Yes
		CVD_COMORB_AIDS	AIDS/Immunodeficiency	Binary	0 = No; 1 = Yes
		CVD_COMORB_DEP	Presence/History of Depression	Binary	0 = No; 1 = Yes
		CVD_COMORB_ANX	Anxiety or Neuroses	Binary	0 = No; 1 = Yes
		CVD_COMORB_PMH	Presence/History of Psychotic Mental Illness (e.g. Schizophrenia)	Binary	0 = No; 1 = Yes
		CVD_COMORB_ABU	Substance Abuse	Binary	0 = No; 1 = Yes
		CVD_COMORB_OST	Osteoarthritis, degenerative arthritis	Binary	0 = No; 1 = Yes
		CVD_COMORB_RHA	Rheumatoid Arthritis	Binary	0 = No; 1 = Yes
		CVD_COMORB_PD	Periodontal Disease	Binary	0 = No; 1 = Yes
		CVD_COMORB_OTH	Other Medical Problems	Binary	0 = No; 1 = Yes
	Lifestyle and Social Factors	SMOK_STAT	Smoking status	Categorical	0 = Current every day smoker; 1 = Current weekly smoker; 2 = Former smoker; 3 = Never smoker; 4 = Others
		ALC_FREQ1	Alcohol Frequency	Categorical	0= Every day/7 days per week; 1= 5 to 6 days per week; 2= 3 to 4 days per week; 3= 1 to 2 days per week; 4= 1 to 3 days per month; 5= Never

CATEGORY	Sub-category	Variable ID	ITEM	TYPE	RESPONSE OPTIONS
		ALC_OCCAS	Alcohol Amount per drinking occasion	Numeric	Average number of units consumed in whole numbers
		PHYS_ACT	Physical Activity	Binary	0 = No; 1 = Yes
Clinician-reported Outcomes Diabetes Control	Glycemic Control	HBA1C	Glycemic Control HbA1c	Numeric	Value of HbA1c
		HBA1C_UNIT	Glycemic Control<br>Units of HbA1c	Categorical	0= mmol/mol; 1= %
		TIR	Glycemic Control Time in range	Numeric	Numerical value of time in range
Clinician-reported Outcomes Diabetes Control	Intermediate outcomes	SBP	Systolic blood pressure	Numeric	Numerical systolic BP in mmHG
		DBP	Diastolic blood pressure	Numeric	Numerical diastolic BP in mmHg
		BL_GLUC	Blood Glucose	Numeric	Value of Blood Glucose
		BL_GLUC_UN	Blood Glucose Unit of Measure	Categorical	0= mmol/mol; 1= %
		LIP_TCHOL	Lipid Profile: Total Cholesterol	Numeric	Value of blood total cholesterol concentration
		LIP_TCHOL_UNI	Units of Total Cholesterol	Categorical	0= mmol/mol; 1= %
		LIP_LDL	Lipid Profile: LDL Cholesterol	Numeric	Value of blood LDL cholesterol concentration
		LIP_LDL_UNI	Units of LDL Cholesterol	Categorical	0= mmol/mol; 1= %
		LIP_HDL	Lipid Profile: HDL Cholesterol	Numeric	Value of blood HDL cholesterol concentration
		LIP_HDL_UNI	Units of HDL Cholesterol	Categorical	0= mmol/mol; 1= %
		LIP_TRI	Lipid Profile: Triglycerides	Numeric	Value of blood triglyceride concentration
		LIP_TRI_UNI	Units of Triglycerides	Categorical	0= mmol/mol; 1= %
		WGT_VAL	Body weight	Numeric	Numerical value of weight
		WG_UNIT	Body weight units	Categorical	1 = kilograms; 2 = lbs
		HGT_VAL_DIA	Body height	Numeric	Numerical value of height
		HGT_UNIT	Body height units	Categorical	1 = centimeters; 2 = inches
		WAISTC	Waist Circumference	Numeric	Value of waist circumference in centimeters
	Hypoglycemia	HYPOL3	Hypoglycemia - Level 3	Categorical	0= 0; 1= 1; 2= 2; 3= > 2
		HYPOL2	Hypoglycemia - Level 2	Numeric	Numeric Response
		HYPOL2_AWAR	Hypoglycemia - Level 2 - Loss of awareness	Binary	0 = No; 1 = Yes
	Micro- and macrovascular complications	MI	Myocardial infarction	Binary	0 = No; 1 = Yes
		CVD	Cerebrovascular Disease - Acute events	Binary	0 = No; 1 = Yes
		LL_AMP	Lower Limb Amputation	Binary	0 = No; 1 = Yes
		LL_AMP_LEV	Lower Limb Amputation Level	Categorical	0= Distal to the ankle joint; 1= Below knee; 2= Above knee
		VIS_THREAT	Visual Outcomes - Diabetes-related Sight Threatening Conditions	Binary	0 = No; 1 = Yes
		VIS_MACULA	Visual Outcomes - Macular edema	Binary	0 = No; 1 = Yes
		VIS_RETINO	Visual Outcomes – Diabetes retinopathy	Categorical	0 = None; 1=Non-proliferative; 2= Proliferative; 3= Unspecified
		AUT_NEU	Autonomic Neuropathy	Binary	0 = No; 1 = Yes
		PERI_NEU_CLI	Peripheral Neuropathy - Clinician Diagnosis	Binary	0 = No; 1 = Yes

CATEGORY	Sub-category	Variable ID	ITEM	TYPE	RESPONSE OPTIONS
		PERI_NEU_PAT	Peripheral Neuropathy - Symptoms experienced by person with diabetes	Categorical	0=None; 1=Numbness; 2= Pain; 3= Paresthesia
		CHARC_F	Charcot's Foot	Binary	0 = No; 1 = Yes
		LL_ULC	Lower Limb Ulcers<br>Active lower limb ulcer present?	Binary	0 = No; 1 = Yes
		PAD	Peripheral artery disease	Binary	0 = No; 1 = Yes
		PAD_PAT	Peripheral artery disease - Symptoms experienced by person with diabetes	Categorical	0= No; 1= Yes, intermittent claudication; 2= Yes, rest pain
		IHD	Ischemic Heart Disease	Binary	0 = No; 1 = Yes
		HF	Heart failure	Binary	0 = No; 1 = Yes
		CHRSTAGE	Chronic Heart Failure<br>Staging	Categorical	0= Stage A (At high risk of HF without structural heart disease or symptoms of HF); 1= Stage B (Structural heart disease but without signs or symptoms of HF); 2= Stage C (Structural heart disease with prior or current symptoms of HF); 3= Stage D (Refractory HF requiring specialist interventions); 4 = Stage unknown; 999= Not assessed
		EGFR	Estimated glomerular filtration rate	Numeric	
		RFTACR	Renal Function Tests/Moderate to Severe Kidney Disease ACR	Categorical	0= ACR <30mg/g or <3mg/mmol; 1= ACR 30-300mg/g or 3-30mg/mmol; 2= ACR > 300 mg/g or >30mg/mmol; 3= ACR unknown
		DIAL_DEP	Dialysis dependent	Binary	0 = No; 1 = Yes
		CD	Cerebrovascular disease	Binary	0 = No; 1 = Yes
		SEX_DYSF	Sexual Dysfunction	Binary	0 = No; 1 = Yes
