# Supplementary figures and images for: Implementing a federated regional diabetes register in a decentralized health system: implications for healthcare organization and the European Health Data Space
Source: Front Public Health. 2026 Jul 16;14:1864673. doi: 10.3389/fpubh.2026.1864673 (PMC13422561; doi:10.3389/fpubh.2026.1864673)

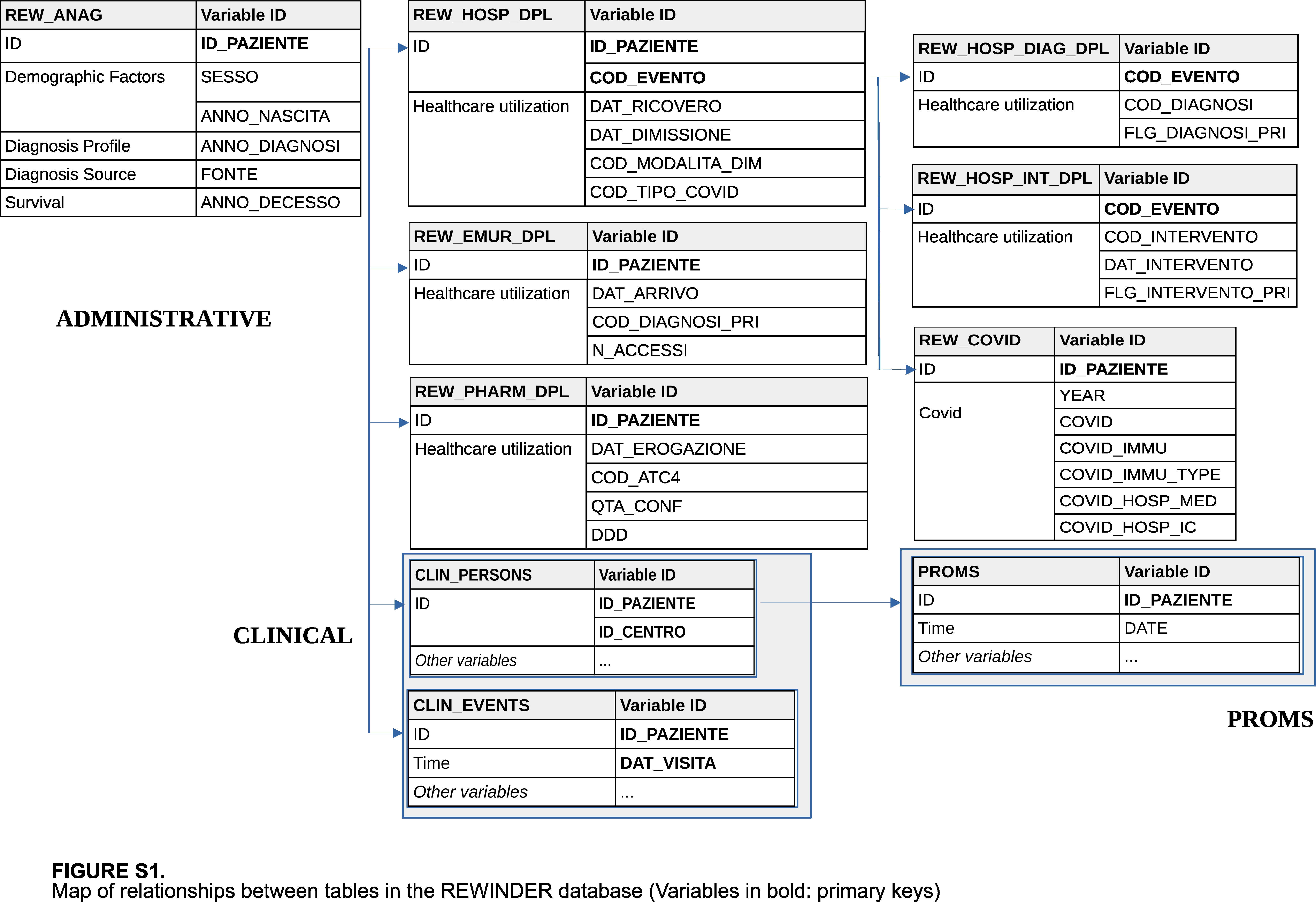

Supplement: Supplementary file 2 [file Image_1.jpg]

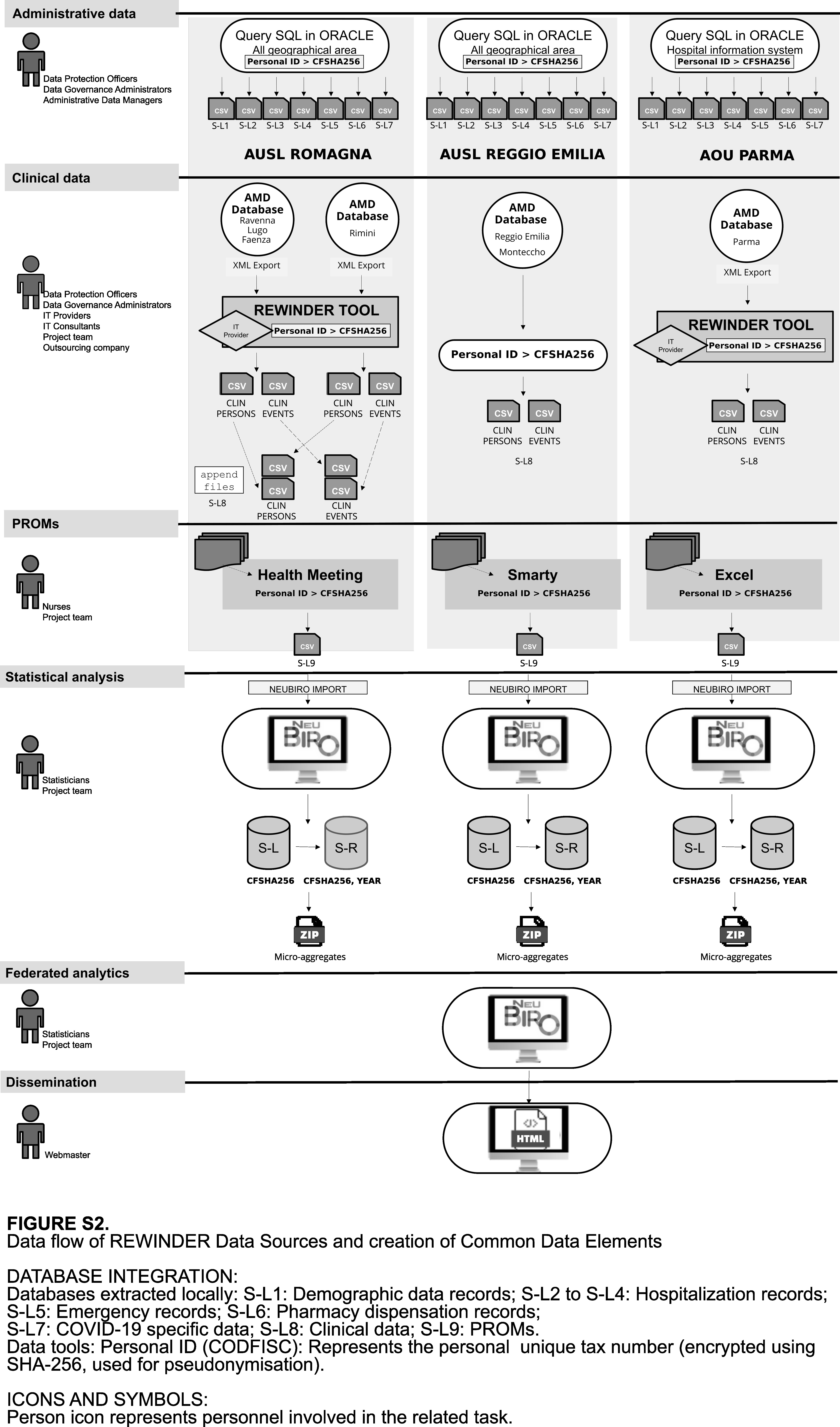

Supplement: Supplementary file 3 [file Image_2.jpg]
